# Recombinant snake antivenoms get closer to the clinic

**DOI:** 10.1016/j.it.2024.03.001

**Published:** 2024-04

**Authors:** Andreas H. Laustsen

**Affiliations:** 1 Department of Biotechnology and Biomedicine, Technical University of Denmark, Kongens Lyngby, DK-2800, Denmark

**Keywords:** Snakebite envenoming, snake venom toxins, antivenom, recombinant antivenom, monoclonal antibodies, broadly-neutralizing antibodies

## Abstract

Snakebite envenomings kill ~100 000 victims each year and leave many more with permanent sequelae. Antivenoms have been available for more than 125 years but are in need of innovation. A new study by Khalek *et al.* highlights broadly neutralizing human monoclonal antibodies (mAbs) that might be used to develop recombinant antivenoms with superior therapeutic benefits.

## Main text

Ever since the emergence of the human race, snakes have been a danger to our kind. They continue to kill ~100 000 victims each year and leave many more with permanent physical and psychological sequelae [1]. Antivenoms based on antibodies (or fragments thereof) isolated from the plasma of immunized animals have been used for over 125 years and have saved countless lives [1,2]. Nevertheless, an unmet medical need remains because these medicines suffer from serious drawbacks relating to their nature and method of manufacture. The key drawbacks include their relatively high cost of production, limited efficacy (especially against non- or poorly immunogenic toxins), and safety concerns relating to their non-human origin which imposes a risk of causing serum sickness or even anaphylaxis in severe cases [3]. For over four decades researchers have been exploring the idea of using mAbs as a substitute for plasma-derived polyclonal antibodies of animal origin [2].

**Figure 1:**
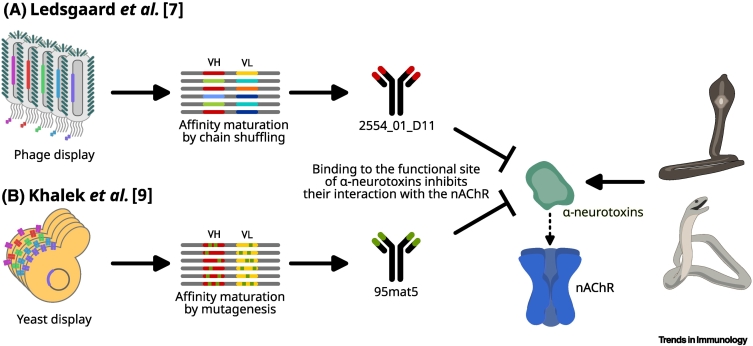
Discovery, screening, and optimization approaches for the development of recombinant broadly neutralizing human monoclonal antibodies (mAbs) that can neutralize long-chain α-neurotoxins from elapid snakes [7,9]. Whereas Ledsgaard *et al.* [7] employed phage display selection and affinity maturation by chain shuffling, Khalek *et al.* [9] utilized yeast display technology combined with affinity maturation by mutagenesis for the discovery of their mAb. Each approach has its own benefits; however, the antibody developed by Khalek *et al.*, 95mat5 [9], seems to better neutralize long-chain α-neurotoxins from different snake species than the antibody 2554_01_D11 used by Ledsgaard *et al.* [7]. Abbreviations: nAChR, nicotinic acetylcholine receptor; VH, immunoglobulin heavy-chain variable region genes; VL, immunoglobulin light-chain variable region genes.

Although some work on the discovery of different mAbs was carried out in the 1980s to 2010s, none of these molecules ever entered into clinical trials, and only a few showed promising effects in neutralizing key toxins [2,3]. Part of the explanation is likely that all the IgG antibodies reported were of non-human (mostly murine) origin and that the human single-chain variable fragments (scFvs) discovered displayed limited therapeutic utility [2,3]. However, in 2013 the first promising camelid mAb fragment (V_H_H) was discovered which could protect mice against a lethal dose of α-cobratoxin from the monocled cobra [4], and in 2018 the first fully human IgG mAbs were reported that neutralized dendrotoxins from the black mamba [5]. From then it took another 4 years before a human IgG mAb could rescue mice from a lethal dose of whole venom from the monocled cobra [6], and it was not until 2023 that a human IgG mAb showed broadly neutralizing capacity against long-chain α-neurotoxins from different elapid venoms (Figure 1A) [7]. These discoveries were all based on phage display technology and deep insights into the composition of snake venoms [2,3,8].

A recent report by Khalek *et al.* now describes the discovery of a new human IgG mAb with superior broadly neutralizing activity against long-chain α-neurotoxins from a range of different elapid snake species (e.g., cobras and mambas) [9]. By using a human fragment antigen-binding (Fab) antibody library combined with yeast display technology, as well as further enhancement of the affinity of the antibody for long-chain α-neurotoxins by using *in vitro* methods and a thorough screening program, the authors identified the human IgG mAb, 95mat5 (Figure 1B). They further demonstrated that this antibody blocks the interaction between selected long-chain α-neurotoxins and the nicotinic acetylcholine receptor (nAChR) *in vitro*, and showed that mice were protected against a lethal venom challenge when the antibody was preincubated with the venom mixture, as well as when the antibody was delivered up to 30 minutes after venom injection. Importantly, this was demonstrated using elapid venoms from three different snake genera (*Naja*, *Dendroaspis*, and *Ophiophagus*) where long-chain α-neurotoxins play an important role in exerting overall toxicity.

To appreciate the achievement of Khalek and colleagues, it is important to understand that snake venoms are very complex mixtures of protein-based toxins, that no two snake venoms share the same composition or even the same toxins, and that the venom of each species contains tens to hundreds of different toxins grouped into over a dozen different protein families [2,3]. Consequently, it is seldom sufficient to neutralize only a single specific toxin to protect a mouse or an individual against a whole venom [5,8]. Being able to neutralize an entire subfamily of toxins is therefore a key step towards the development of fully recombinant antivenoms based on carefully designed oligoclonal mixtures of broadly neutralizing antibodies [5,7]. Finally, Khalek and coworkers also succeeded in co-crystallizing their antibody 95mat5 with a recombinant long-chain α-neurotoxin, enabling elucidation of the structure, and they concluded that toxin neutralization is achieved by receptor mimicry in which the paratope of the antibody adopts a conformation similar to that of the nAChR, allowing the toxin to bind to the antibody via its functional site [9]. Although this finding may seem logical, it is noteworthy that the mechanisms of action behind the neutralization of snake toxins are poorly understood, and such structural insights may significantly aid in the development and optimization of broadly neutralizing antibodies against other neurotoxins, as well as against other medically important snake toxin families.

In addition to previous reports of human mAbs that can protect rodents against snake toxins and whole venom [6,7], the study by Khalek *et al.* [9] represents an important step towards consolidating how recombinant antivenoms might be designed. However, other obstacles need to be overcome before recombinant antivenoms can enter clinical development. An important challenge is to achieve a better understanding of how oligoclonal antibody mixtures should best be designed to (i) robustly neutralize more complex whole venoms from different snake species, (ii) neutralize toxin subfamilies other than/in addition to long-chain α-neurotoxins, and (iii) account for intraspecies venom variation which may further complicate the design of broadly neutralizing recombinant antivenoms [8]. Another challenge will be to determine how to manufacture recombinant antivenoms based on mixtures of mAbs in a cost-competitive manner that is compatible with deployment of the end antivenom products in low- and middle-income regions with limited healthcare infrastructure [3]. Finally, some snake toxins exhibit surprising pharmacology in mice when bound to mAbs, and the administration of the latter can even give rise to antibody-dependent enhancement of toxicity (ADET) [10], thereby worsening the effect of the toxin rather than neutralizing it. This calls for caution and suggests that additional antibody engineering might be necessary to successfully develop neutralizing mAbs against some snake toxin families.

The study by Joseph Jardine and colleagues [9] thus represents a significant contribution to the field of antivenom research; it may even place the discovery of recombinant antivenoms in the same arena as the discovery of mAbs against infectious diseases. The discovery phase no longer represents a serious bottleneck, but challenges relating to low-cost manufacturing, clinical trial design, and attractive business models are still a hindrance to bringing antibody-based therapies to the market.
